# From Fish Waste to Value: An Overview of the Sustainable Recovery of Omega-3 for Food Supplements

**DOI:** 10.3390/molecules26041002

**Published:** 2021-02-13

**Authors:** Vincenzo Gabriele Alfio, Cosimo Manzo, Raffaella Micillo

**Affiliations:** Avantech S.R.L., Via Quarto dei Mille 6, I-90129 Palermo, Italy; gabriele.alfio@gmail.com (V.G.A.); manzo@avantech.it (C.M.)

**Keywords:** omega-3, fish waste, supplements, fish oil extraction, PUFA, EPA, DHA, supercritical fluid extraction, biorefinery

## Abstract

The disposal of food waste is a current and pressing issue, urging novel solutions to implement sustainable waste management practices. Fish leftovers and their processing byproducts represent a significant portion of the original fish, and their disposal has a high environmental and economic impact. The utilization of waste as raw materials for the production of different classes of biofuels and high-value chemicals, a concept known as “biorefinery”, is gaining interest in a vision of circular economy and zero waste policies. In this context, an interesting route of valorization is the extraction of omega-3 fatty acids (ω-3 FAs) for nutraceutical application. These fatty acids, such as eicosapentaenoic acid (EPA) and docosahexaenoic acid (DHA) have received attention over the last decades due to their beneficial effects on human health. Their sustainable production is a key process for matching the increased market demand while reducing the pressure on marine ecosystems and lowering the impact of waste production. The high resale value of the products makes this waste a powerful tool that simultaneously protects the environment and benefits the global economy. This review aims to provide a complete overview of the sustainable exploitation of fish waste to recover ω-3 FAs for food supplement applications, covering composition, storage, and processing of the raw material.

## 1. Introduction

Food of both plant and animal origins represents a considerable source of compounds relevant for human health. Bioactive substances can also be recovered from waste and byproducts of the food supply chain thus contributing to the implementation of sustainable practices in waste management [1].

According to a commonly accepted hierarchy of solutions in waste management, the prevention of waste generation should be the primary solution to be implemented. Alternative actions to be taken include the exploitation of waste for the retrieval of added-value products, an extremely attractive possibility both from an environmental and economic point of view. The actions fit into the overall definition of “biorefinery”, which is the sustainable conversion of biomass into energy and other biobased products [2,3].

Even if data on food waste amounts are somehow affected by the lack of a generally accepted definition of what is considered “waste”, it is estimated that around one-third of food is discarded [2].

A considerable contribution to global waste is constituted by fish leftovers and processing byproducts that represent from 20 to 80% of the original fish and include a wide range of materials like damaged whole fish, and non-edible parts like viscera or skin [4,5]. All these discarded materials have attracted attention over the last decades, triggering researchers to develop methods to process them into useful products, making the most of their biochemical heterogeneity: lipids, proteins (or their hydrolysates), and polysaccharides are just some of the molecules that can be obtained.

Common applications of fish leftovers include but are not limited to animal feed, production of energy through biodiesel and biogas, natural pigments (e.g., astaxanthin or βcarotene), soil fertilizers, food packaging, and enzyme isolation [6,7].

Recovery of marketable byproducts from fishery waste represents a paramount strategy to reduce disposal costs, benefit the environment, and generate additional revenue. Additionally, the recovery of fish oil from waste may diminish the pressure of industrial fishing on fish stocks [8]. In this frame, an interesting route to the valorization of rest raw materials and waste is the isolation of ω3- FAs such as docosahexaenoic acid (DHA, 22:6ω-3) and eicosapentaenoic acid (EPA, 20:5ω-3), which are widely distributed in marine sources like fish and other seafood.

EPA and/or DHA are claimed to have a preventive role in the development and progression of a broad range of conditions, including cardiovascular diseases (CVDs), neurological diseases, infant development, and cancer [9,10,11]. Despite evidence for ω-3 FAs effectiveness is not always conclusive, their benefits are reported also based on some randomized clinical trials, and the interest in their protective role is considerable [9].

Fish consumption should be preferred to ω-3 FAs supplements because it provides beneficial nutrients not contained in fish oil. Nevertheless, when sufficient intake is impossible, supplementation is reasonable and sometimes recommended [12].

Traditional sources of ω3-FAs are wild and aquacultured fish but, due to the increased demand, the need for alternative and compatible sources is dramatic [8]. Projections show that in 2025 fish oil for human consumption will reach 771,000 tons [13].

In accordance with the circular economy and the zero-waste concepts [14], not only is a sustainable extraction and purification of ω-3 FAs from fish waste desirable, but it should be strongly encouraged since all the byproducts of the process can be turned into valorized products: protein moiety as animal feed and source for enzymes and bioactive peptides, glycerol for the production of valuable chemicals short-chain unsaturated fatty acids (FAs) as liquid biofuel, fish bones as a source of minerals, collagen, and gelatin [7,15]. Waste valorization should be carried out minimizing the environmental impact, by ensuring that the whole process of recovery provokes a lower impact than other waste management practices.

In this review, several issues affecting the production of ω-3 FAs supplements from fish waste material will be discussed, including waste composition, storage, and processing, focusing on the sustainability of the processes and providing a basic overview of the potentialities of supercritical fluids in different steps of the supplement production. To the best of our knowledge, this is the first review covering the whole process from waste to ω-3 FAs formulations for supplement applications. The main aim is to provide the interested reader with a wide and complete overview of the topic and to improve the sustainable exploitation of this valuable resource.

## 2. Lipid Content in Fish Oil

**Figure 1:**
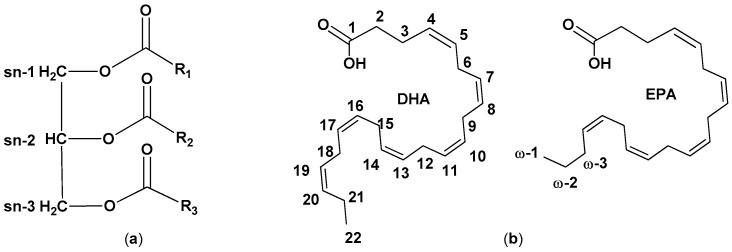
(**a**) Simplified structure of a triacylglycerol and (**b**) chemical structure of and docosahexaenoic acid (DHA) and eicosapentaenoic acid (EPA).

Lipids are mainly present in fish oil as triacylglycerols (TAGs), fatty acid esters of glycerol. Glycerol carbon atoms are numbered according to a stereochemical system, positions sn-1 and sn-3 being the outers, position sn-2 the central one (Figure 1). Considering that FAs can be differently arranged in TAGs, if n is the number of different FAs in a TAG, n^3^ different molecules (including enantiomers) are possible, giving each oil its unique and complex composition [16]. A minor number of FAs occurs as phospholipids, amphipathic molecules containing polar head groups linked to the glycerol moiety via phosphate linkage (Figure 1). Examples of polar head groups are choline, ethanolamine, and inositol [17].

**Figure 2:**
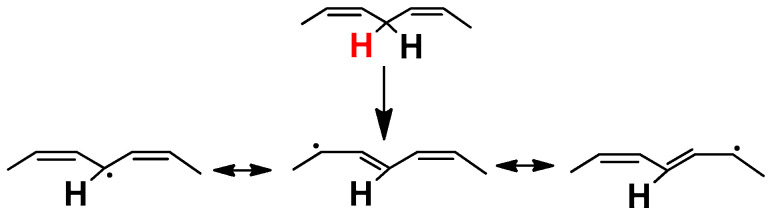
Rearrangement of the 1,4-pentadiene structure to give a stabilized conjugated diene.

Different classes of FAs are present in lipids: saturated FAs (SFAs), monounsaturated FAs (MFAs, containing one C-C double bond), and polyunsaturated FAs (PUFAs, containing more than one C-C double bonds). Most natural unsaturated fatty acids’ double bonds show a *cis* configuration that confers them a kinked shape and impairs the lipid packing, resulting in lower melting points and increased membrane fluidity (Figure 2). FAs are classed according to the position of the first double bond counting from the terminal omega (ω) carbon: ω-3 FAs have the first double bond on carbon 3 while ω-6 FAs have the first double bond on carbon 6 [13,15,16,17,18].

Broadly speaking, the lipid composition of fish widely varies according to geographic location, type of water (marine or freshwater), feed, season, and maturity of the fish [18]. Additional differences may be caused by post-capture handling and processing. Food availability is a major determinant of fish fatty acids composition since fish are not able to produce ω-3 FAs but accumulate them from algae, plankton, and prey fish [8]. Accordingly, EPA and DHA content is higher in marine fish than in freshwater fish because marine plankton presents a high level of ω-3 PUFAs [19,20]. Farmed fish may be characterized by a higher content of DHA and EPA depending on their feed (typically fishmeal or fish oil) [8]. In spring and summer food is usually more available than in other seasons so fat storage increases [18,19].

Membrane and storage lipid composition also varies with thermal changes: cold is usually associated with an increase in unsaturation to maintain membrane fluidity and functionality at low temperature [8,21]. Finally, sexual maturity also affects lipid content that decreases during the spawning period due to the higher consumption of fat reserves [18].

All these factors must be taken into account when choosing a waste source for extraction of ω-3 fatty acids and explain why data on fish FAs composition are not always similar [19]. For example, considering the fatty acid profiles of thirteen different seafood species caught in the Northeastern Mediterranean coast, SFAs range from 27.68 to 36.59%, MUFAs from 8.99 to 35.84%, and PUFAs from 10.69 to 39.57%. FAs typically detected are myristic acid (C14:0), palmitic acid (C16:0), palmitoleic acid (C16:1ω7) heptadecanoic acid (C17:0), stearic acid (C18:0), vaccenic acid (C18:1ω-7), oleic acid (C18:1ω-9), linoleic acid (C18:2ω-6), arachidonic acid (C20:4ω-6), cis-5,8,11,14,17-EPA (C20:5ω-3) and cis-4,7,10,13,16,19-DHA (C22:6ω-3) [22]. Fish oil can contain up to ca. 50% of omega-3 PUFAs, with EPA and DHA accounting from 5 to 26 w/w% of total FAs [20,23].

Fish lipids are currently being extracted from a wide range of species, including cod, herring, mackerel, tuna, salmon, anchovy, menhaden, and sardine [16]. Byproducts availability varies within species and processing methods, but usually includes viscera, skin, heads, bones, and fins. The oil distribution in the tissues is usually heterogeneous. For example, mackerel’s skin, which is usually discarded during processing, has a higher content of PUFAs if compared with viscera and muscle [20]. Livers and viscera of little tuna (*Euthynnus alletteratus*) contain a higher lipid amount than the other organs [21]. Tuna heads are a common raw material but their cooking yields a dark and malodorous oil, which requires several refinery processes [24]. Tuna eyeballs that are separated from tuna heads before cooking could render higher quality fish oil rich in ω-3 FAs (32.72% DHA and 7.07% EPA, with respect to total PUFAs) reducing the number of steps in the refinery process [24,25].

Knowing the composition of raw material is essential to optimize extraction and purification processes in terms of yields and quality of the oil. However, consideration should be given not only to fatty acid content but also to local availability.

## 3. Omega-3 Fatty Acids and Health

DHA and EPA are produced by krill and fish through bioconversion of FAs accumulated by consuming zooplankton (fish and krill) or phytoplankton (krill) [26].

EPA and DHA can also be produced by the human body converting α-linoleic acid (ALA), an essential FA that must be obtained from other organisms that can synthesize it, typically plants. However, due to low conversion efficiency, food intake is the principal way to increase their level in the human body. After absorption, PUFAs are incorporated at a cellular level into triglycerides, phospholipids, and cholesterol esters [14].

Omega-3 FAs play both a structural role as components of cell membranes (regulating their architecture and permeability) and a metabolic role (providing energy for the body). Moreover, they are used to produce eicosanoids, oxygenated metabolites involved in many cell-signaling pathways regulating the modulation of cardiovascular, pulmonary, reproductive, immune, and endocrine systems. Some examples are prostaglandins, thromboxanes, and leukotrienes [17]. The ω-6/ω-3 fatty acids ratio also seems to have important implications for the development of some chronic diseases but the optimum relative intake is not still clear.

The potential health benefits of ω-FAs consumption are related to a wide range of pathologies, including cardiovascular disease (CVD), infant neurodevelopment, cancer, Alzheimer’s disease, cognitive function, rheumatoid arthritis, depression, attention deficit hyperactivity disorder (ADHD), and some eyes conditions like age-related macular degeneration, and dry eyes [10,11,27,28,29,30,31,32,33,34].

These observations stem from numerous observational and prospective studies. However, caution must be exercised when trying to undoubtedly correlate a benefit to a specific bioactive compound (in this case, ω-3 FAs in seafood). Other aspects should be taken into account, including the presence of other nutrients, the substitution of seafood for other less healthy foods, a better social status, or a health-conscious lifestyle. Besides, a combination of different factors cannot be ruled out. The availability of data from randomized clinical data is essential but unfortunately these data are often conflicting and not conclusive. This may be ascribed not only to the different doses used in different studies but also to differences in population, diet, therapies, or statistical analysis. For example, some prospective studies relate a higher intake of omega-ω-3 fatty acids with a lower risk of breast and colorectal cancer, but genetic risk and gender seem to play an important role requiring additional research [35,36].

Maybe the more controversial subject is the relationship between ω-3 FAs intake and CVDs, disorders of the heart and blood vessels, which include coronary heart and cerebrovascular disease [9,10,37,38].

Some observational studies correlate higher dietary or plasma levels of ω-3s FAs with a lower risk of CVDs [37,39]. Looking at some clinical trials data from 1968 to 2019 [9], EPA and DHA are reported to induce a slight but significant reduction in serum triglyceride levels, rate of cardiovascular mortality, and rate of coronary heart disease events [9,38], but results are often conflicting with the omega-3s sometimes not provoking any significant effects [40,41].

Overall, since evidence exists that EPA and DHA have a positive effect on serum triglycerides levels and heart health, causing a slight but significant reduction of coronary death and coronary events, ω-3 FAs intake is generally recommended (with some differences in public health advice across countries) [9]. Getting ω-3 FAs directly through fish consumption is usually preferable. However, when it is impossible to get enough ω-3 FAs exclusively through the diet, supplementation may represent an effective option (that should always be discussed with a doctor) [9]. Supplements may also offer a solution to some downsides of seafood consumption, i.e., the potential concurrent intake of environmental pollutants such as dioxins or heavy metals, which could compromise the health status of people consuming contaminated fish regularly because of bioaccumulation].

## 4. Omega-3 Fatty Acids Recovery from Fish Waste: Some Critical Issues

When considering the whole process that leads from fish waste to ω-3 FAs formulations, some inherent critical issues and technical challenges regarding the processing of this raw material must be taken into account. These include degradation phenomena and the possible presence of contaminants that may be coextracted.

Waste from the food supply chain is characterized by an elevated content in water and nutrients and it is, in general, a good growth medium for bacteria and fungi that can be toxic and/or deteriorate active compounds at room temperature and in a short time, impairing their nutritional value.

Microbial spoilage under anaerobic storage conditions can cause fish waste deterioration resulting in a complex mixture of chemical products like volatile nitrogen or sulfur compounds (e.g., ammonia or hydrogen sulfide).

The high content in PUFAs makes fish waste highly susceptible to lipid peroxidation, a complex oxidative process that proceeds by a free-radical mediated chain reaction and involves three main steps: initiation (or induction), propagation, and termination.

For the chain reaction to start, the presence of a trigger like heat, light, metal, peroxide, or hydroperoxide is necessary. The reaction is initiated by the abstraction of a hydrogen atom followed by the addition of an oxygen radical. In general, PUFAs consist of 1,4-pentadiene structures (Figure 2) that are particularly sensitive to lipid peroxidation: the methylene hydrogens are weakly bonded to the C atom and are easily abstractable. The removal of a hydrogen atom at the bisallylic position leads to the formation of an alkyl radical (L^•^) that is stabilized by a molecular rearrangement of the double bond to form a conjugated diene (CD). CDs absorb UV radiation and can be monitored by absorbance at 234 nm [42].

Dienes can combine with oxygen to form a peroxyl radical (LOO^•^) creating hydroperoxides (LOOH) and other radicals, thus propagating the reaction (autoxidation). Hydroperoxides can eventually split into alkoxy radicals (LO^•^) and hydroxy radicals (^•^OH), generally considered primary products of lipid peroxidation. This chain of reaction can be schematized as follows:

LH → L^•^ + ^•^H

L^•^+ O_2_ → LOO^•^

LOO^•^ + LH → LOOH + L^•^

LOOH → LO^•^ + ^•^OH

These products can abstract an electron from an adjacent covalent bond breaking the aliphatic chain (β scission) and forming secondary products, including aldehydes and ketones, responsible for the bad smell and off-flavors of rancid oil.

A complex mixture of volatile and non-volatile compounds with different molecular weight, polarity, and functional groups is formed [43]. The increase in double bonds increases the sites available for radical attack; when radicals accumulate, they can interact creating stable non-radical products and terminating the reaction.

The high heterogeneity of lipid oxidation products makes their evaluation and measurement quite complex. In general, fish oils and fish oils ethyl esters shall comply with some oxidation parameters (Table 1), reported by FAO Standard for fish oils [44]:

The acid value of an oil depends on its content in FFAs that can promote oxidation and alter the oil’s organoleptic properties.

The peroxide value (PV) measures primary oxidation products by titration, while secondary non-volatile oxidation products (e.g., 2-alkenols and 2,4-alkadienals) are determined by their reaction with *p*-anisidine (pAV). Since a colorimetric method is used to determine pAV, this test is not suitable for colored oils absorbing at 350 nm due to interference phenomena. Moreover, since *p*-anisidine reacts with aldehydes, this test is unsuitable for flavored oil because they are often added with aldehydes containing additives.

These parameters account for the progression of the oxidation process. Peroxides are the first compounds created so an increase in PV is initially observed. As the oil oxidizes, peroxides react to give secondary products, so PV decreases, and pAV increases. The ToTox value (“total oxidation of the oil”) takes into account both primary and secondary oxidation products and is calculated as follow:

ToTox = (2 × Peroxide value) + (1 × Anisidine value)(1)

Other tests that are used for determining the oxidative quality of oils include the spectrophotometric measurement of conjugated dienes or the determination of thiobarbituric acid reactive substances (TBARS) by the TBARS assay that measures the malondialdehyde (MDA) and its related compounds. Interpretation of data from this assay requires caution because prolonged heating can cause the evaporation of volatile secondary oxidation products resulting in a decrease in TBARS.

Some consequences on the quality and nutritional values are the decrease in caloric content and the loss in essential fatty acids and lipid-soluble vitamins. Lipid peroxidation can damage cell structures, impact membrane functionality, and is involved in the toxicity process that leads to cell death. Moreover, some oxidation products are related to aging, mutagenesis, and carcinogenesis. For example, aldehydes such as MDA can bind to proteins and nucleic acids [43]. However, clinical trial results are not always conclusive, and potentially harmful effects may vary with dose, actual local concentration, human metabolism, and antioxidant defenses. In addition, some oxidation products from ω-3 FAs may be beneficial to health [46].

Oxidation usually causes the darkening of oils, off-flavors, and pronounced unpleasant odors caused by the mixture of volatile secondary products, thus affecting also customers’ acceptability [43]. Fat deterioration can also be due to lipases, enzymes that may cause extensive autolysis, splitting free fatty acids from the glycerol, impacting negatively both the yield and the quality of the oil [47].

The low oxidative stability poses a serious problem that covers several aspects from storage to production, affecting shelf life, nutritional value, safety, and acceptability of products among consumers [43]. All these issues require special handling and compel the industry to seek timely and proper storage and processing strategies.

Light and oxygen exposure, heating, and pro-oxidants, such as lipoxygenases, singlet oxygen, and transition metals accelerate lipid oxidation. Precaution should be taken during the whole processing: waste should be immediately frozen and stored in glass or Teflon sealed containers, trying to minimize air contact. Freezing damages tissue because osmotic shock and crystal formation break membranes that separate lipids from lipolytic enzymes that are still active at −20 °C, so lower temperatures are needed [42]. Fish oil should be stored at −20 °C in amber containers to shield it from light and, possibly, under a nitrogen atmosphere. Both in the case of fish waste and oil, freezing and thawing cycles should be avoided.

Prolonged heating of raw material improves extraction yields but promotes oxidative degradation: secondary oxidation products may react with amino acids via Maillard reaction giving dark polymers [24]. Additionally, high temperature increases lipolysis: free fatty acids are more reactive than TAGs (that are more sterically hindered) and the rate of oxidation increases.

All these precautions are sometimes insufficient for effective protection and make it necessary the addition of natural or synthetic antioxidant compounds that prevent or retard oxidative deteriorative processes by different mechanisms, including radical and/or oxygen scavenging and metal chelation.

Natural antioxidants (e.g., tocopherols) protect to a certain extent fish oil but additional supplementation is usually advisable. Antioxidants currently used in food are, for example, tocopherols (up 6000 mg/Kg singly or in combination) or synthetic additives such as 2,6-di-tert-butyl-*p*-cresol (or butylated hydroxytoluene o BHT, up 200 mg/Kg) [44,48].

Caution should be exercised because antioxidants could be accidentally removed during processing and should be reintroduced. Additionally, a proper packaging methodology involving a modified atmosphere depleted of oxygen may improve oil stability [43].

Another possible option is microencapsulation that can shield lipids from light, heat, and oxygen, protecting the oil from degradation and improving storage stability [42]. Different encapsulation techniques have been devised to protect and deliver ω-3 FAs and some of them will be discussed further.

Marine pollution has resulted in the presence of potentially toxic substances such as heavy metals (and metalloids), pesticides, polychlorinated biphenyls (PCBs), and dioxins in fish oils [49]. The actual concentrations observed depend not only on the species but also on the environmental contamination of their natural habitats. These fat-soluble contaminants accumulate and have a huge impact especially when considering oil from predatory fish, on the top position of the food chain.

Heavy metals such as mercury, cadmium, lead, and arsenic pose a threat to human health causing several adverse effects on a number of organs [50]. Seafood consumption is one of the main routes of exposure to heavy metals that accumulate in fish tissues and this could jeopardize its benefits. The metal amount in seafood does not necessarily pose a significant health risk and consuming a diversity of fish could limit potential harms, nonetheless, care must be taken when dealing with fish derivatives to avoid their concentration in the final product.

Dioxins and PCBs are known to be carcinogenic to humans and have been related to reproductive disorders, neurological problems, and other diseases [8].

Due to their persistence in the environment and accumulation over time, harmful effects may be visible only after long-term fish oil consumption. Similar to metals, these contaminants can be transferred to the oil due to their lipophilicity and undergo concentration.

To maximize health benefits and minimize potential detrimental effects, extraction, and purification processes should reduce contaminants in the final product without impacting the quality of the oil [8]. Attention should be also paid to the quality of fish oil as a feed for aquaculture, to avoid further bioaccumulation.

Refining processes get rid of most undesired components or reduce them to an acceptable level for human consumption. Neutralization, deodorization, and bleaching remove metals and persistent organic pollutants while molecular distillation is effective at removing halogenated compounds. These techniques operate sometimes under conditions that can alter the quality of the oil, promoting its oxidation. Treatment with activated carbon absorbs dioxins and PCBs under milder conditions without affecting DHA and EPA content.

Decontamination efficiency is variable and monitoring these contaminants in fish oil supplements is recommended to evaluate their possible contribution to total human exposure.

## 5. Fish Oil Extraction

The fish raw material consists of three main fractions: solid (free fat dry matter), oil, and water. Numerous apparatuses and techniques have been developed to extract fish oil, to improve both yield and quality. Hundreds of experimental articles, reviews, and patents have been published not only describing technical issues but also drawing attention to the profitability of the process, which should be ideally easily scalable on an industrial scale [51].

Traditional lipid extraction techniques are based on their hydrophobicity and involve the use of organic solvents like hexane, petroleum ether, methanol, and chloroform [52,53]. The choice of the solvent is affected by many factors: low boiling point (to ease the recovery of the product), cost, availability, flammability, disposal procedures, and toxicity. The extraction efficiency is high but due to the restriction in using organic solvents in the food industry and to the research of environmentally friendly and sustainable processes, they are currently used on a laboratory scale, often for comparative or analytical purposes. A classic method is described by Bligh and Dyer in 1959 [54].

To reduce time and use of solvents it is possible to use the Soxhlet extractor, an apparatus that operates recycling a minor amount of solvent ensuring continuous contact between waste and solvent. Despite its efficiency, a shortening in time, and a decrease in solvent consumption, the drawbacks of solvent extraction are not completely overcome. To further reduce costs and time, some variations assisted by microwaves or ultrasounds are possible and will be discussed in the next section [1].

An interesting yet emerging strategy to replace classic organic solvents was proposed by Ciriminna et al. in 2019: limonene from waste orange peel was used to extract ω-3 FAs from anchovy filleting leftovers by maceration under mechanical stirring at room temperature. The solvent can be removed under reduced pressure and recycled in consecutive extraction cycles. The two major costs of this process are the labor and the electricity needed to recover the solvent [55,56].

On an industrial scale, fish oil extraction is commonly carried out by a process known as “wet reduction” (or “wet rendering”), which avoids the impact of traditional methods with organic solvents. This process is described in detail by the Food and Agriculture Organization of the United Nations [47]: although outdated, this document offers an interesting and informative description of the method.

The process aims to separate the three fractions through several unit operations, including:

- Cooking;
- Pressing/Centrifugation;
- Decantation;
- Drying.

**Figure 3:**
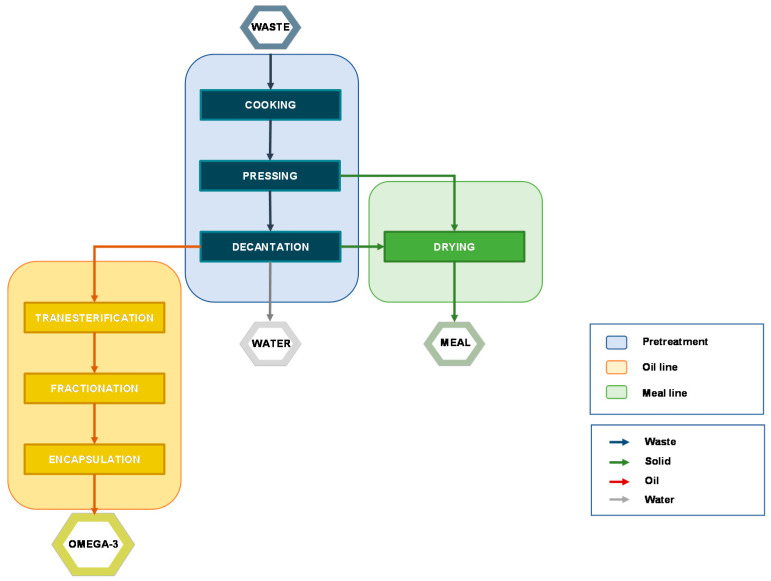
Block flow diagram (BFD) of a typical process from fish waste to final products.

Figure 3 shows the complete block flow diagram (BFD) of a typical process that leads from the waste to the final products, but some alternatives may occur. The layout of the plant depends on different parameters including the equipment for each operation, energy-saving facilities, automation, environmental protection, and raw material content in terms of dry matter, protein, and fat. In particular, if the fat content is less than 3%, the recovery of oil could not be economically convenient [47].

Cooking—High temperature coagulates protein and induces the rupture of the cell walls, releasing oil and water. This step is crucial for a successful oil extraction. Different ranges of temperature and time have been investigated pointing out that cooking at 90–100 °C for 15–20 min allows achieving optimum results. It is noteworthy that control of the heating temperature and time is essential to avoid an overcooking that may affect the compressibility and prevent effective evaporation of water due to the presence of suspended solids. Furthermore, the extreme temperature may induce the degradation of thermolabile compounds via lipid peroxidation [43]. According to the characteristics of the raw material, different conditions and equipment may be required so, practical experience is paramount to obtaining a high-quality product [47].

Pressing—The sludge resulting from the cooking step is pressed to remove most of the solid that is used to produce fishmeal. To achieve a good separation the material should present an appropriate grade of porosity to make the liquid flow more easily through the solid. Two fractions are obtained: press liquor (oil, water, and some solid residues) and press-cake (wet solid). A quicker and more controllable separation is achieved by centrifugation, which is preferred when the solid content is high. A drawback to this operation is the production of emulsions that may hinder the following operations. Besides, the higher content in residue moisture requires an increase in fuel consumption [47].

Decantation—The separation of the press liquor may be carried out by decantation or centrifugation, both based on the specific gravity of each component. Decantation is an inexpensive but slow operation that consists of leaving the press liquor in a tank until the three layers are separated. This solution usually does not allow high yield and has been overtaken by centrifugation that speeds up the process improving the quality of the product but it is still adopted in small plants because a centrifugation apparatus could be not economically justified.

Drying—The press-cake is unstable due to the content in moisture that should not exceed 12% (recommended moisture content in animal feed) [8]. The solid is disintegrated, mixed, and heated at high temperature (ca. 100 °C) to ease the removal of water and prevent microbial activity, killing bacteria that may be present (e.g., *Salmonella*). Higher temperatures should be avoided because they may alter the nutritional value and induce lipid peroxidation as well. The cost of drying is affected by the content in water: since fat and water content are inversely related, a high fat/water ratio not only will improve oil yield but would also reduce drying energy [47]. The resulting proteinaceous flour-type material is used as animal feed.

## 6. Alternative Methods for Oil Extraction

Despite avoiding the use of organic solvents, wet rendering is an energy-intensive process and may prompt the degradation of heat-sensitive PUFAs. Consequently, alternative methods are sometimes considered to extract fish oil from waste. These include microwave-assisted extraction (MAE), ultrasound-assisted extraction (UAE), enzymatic methods, and the use of supercritical fluids (SFs) [57].

MAE uses microwaves to warm solvents in contact with the biomass reducing its moisture and resulting in increased pressure that disrupts the cell membranes of the fish. The extraction process is sped up, lower temperatures are required, and automatization is possible, but the heat generation may induce oxidative degradation. The extraction efficiency is affected by the time and frequency of microwaves, time, and sample particle size. This method is not commonly used for fish oil extraction but some studies show that the efficiency is similar or greater than traditional techniques [57]. A green solvent such as water can be used, making this method attractive from an environmental point of view [58].

UAE exploits acoustic cavitation to break the cell walls: the mechanical impact improves the penetration of the solvent and eases the release of the oil. Advantages are decreased extraction time and solvent consumption while the major drawbacks are the high power consumption and the difficulty of scale-up [57]. The number of publications about the application of this method in fish oil extraction is scarce, but yields seem to be comparable or higher than those of conventional methods [59,60].

An interesting option that requires neither organic solvents nor high temperature is the enzymatic extraction that involves the use of exogenous proteolytic enzymes (e.g., protease, exopeptidase, or endopeptidase) to remove lipids from fish waste [61,62]. Several low-cost commercial enzymes are available but alcalases seem to extract the highest amount of fish oil. Other parameters to be considered are enzyme concentration, pH, reaction time, and temperature. An increase in the enzyme concentration improves the extraction provided that there is substrate available to bind to. The optimum pH depends on the enzyme but in general extreme values can induce enzyme denaturation and must be avoided. Low temperatures usually do not provide enough energy to promote the collisions between the enzyme and the substrate and favor the binding. Even if an increase in temperature speeds up the reaction, extreme temperatures inactivate the enzymes due to their denaturation. Three phases are obtained: a heavy phase on the bottom (mainly containing polar lipids), an intermediate phase containing water-soluble material, and a light oily phase enriched in neutral lipids. These methods are in line with green chemistry practices: enzymes are reusable, there is no need for toxic solvents or extreme temperature conditions, and byproducts production is limited. It is possible to obtain ω-3 FAs enriched oil with low energy requirement and low investment for a large scale process [51]. However, the oil usually needs further treatments to remove some undesired components and the scale-up may be difficult [57].

A supercritical solvent is a fluid that under specific conditions of temperature and pressure (in the region above its critical point) shows unique properties: density-wise it is similar to a liquid while its viscosity, diffusivity, and compressibility are similar to the gas ones. Since the polarity of SFs increases with density, and because of the variation of density with pressure and temperature, tuning these two parameters modulates SCF solvent power allowing the extraction and/or concentration of a wide range of substances [51]. The most used SCF is scCO_2_ because its critical conditions are relatively easy to access (Tc = 304.15 K and Pc = 7.38 MPa). It is considered safe because non-toxic, non-corrosive, and non-flammable. Moreover, it can be easily removed after the extraction because it is gaseous and leaves no traces in the product. Operating at low temperatures, this technique is suitable for thermolabile compounds, while the displacement of the oxygen by the scCO_2_ contributes to preserving ω-3 FAs from oxidation [63].

When dealing with supercritical fluid extraction (SFE), pretreatment of the waste is important because it defines water content and particle size. Water hampers CO_2_ diffusion and affects oil solubility in scCO_2_, so often waste is freeze-dried before extraction. Despite the energy requirement, the extraction yield is significantly improved. Particle size, on the other hand, affects extraction yields, limiting mass transfer, and causing channeling phenomena [63].

SFE is useful when processing raw material with low oil content because cooking causes the formation of a stable water/oil emulsion that is difficult to separate. It achieves similar or better yields in comparison with other methods, prevents lipids oxidation, significantly reduces some kinds of pollutants [51]. Despite being a promising method, it has not replaced the wet rendering method on an industrial scale, partly due to the high initial investment. The cost could be compensated by the elevated yields and the high selectivity, which reduces the need for extensive refining. Moreover, it should be considered that the power consumption might affect the sustainability of the process if the energy is not supplied exploiting renewable resources.

## 7. Fish Oil Refining

The production of oil suitable for food consumption requires the removal of undesired compounds that may affect consumer acceptability (e.g., smelly or colored substances) and sometimes be toxic (e.g., heavy metals, dioxins, or polychlorinated biphenyls) [50,51]. Steps that are usually included are the following: degumming, neutralization, bleaching, and deodorization [60,64].

The degumming removes hydratable and non-hydratable phospholipids from the oil. The former are separated through the addition of water that makes them insoluble in the oil and removable by filtration or centrifugation (water degumming). The latter are present as calcium or magnesium salts of phosphatidic acid and are separated by treating the oil with phosphoric or citric acid that converts them into the hydratable form. This treatment also positively affects the stability of the oil because removes part of the metals that can act as a pro-oxidant.

The neutralization is the clearing of free FAs and residual phospholipids by reaction with alkalis (typically soda) to reduce oil acidity. Free fatty acids are converted into soaps that can be easily separated by centrifugation due to their insolubility in oil. Alkalis also react with some of the pigments that cause unpleasant colors, improve the aspect of the oil.

The bleaching is the absorption of pigments, trace metals, oxidation products, and other contaminants using activated earth or carbon that should be carefully eliminated by filtration because may content residual metals that can act as pro-oxidant.

Deodorization is the removal of smelly compounds and it is usually carried out by steam distillation at high temperature (190–210 °C). Since at temperatures above 180 °C lipid peroxidation occurs, the process may be performed under reduced pressure or nitrogen atmosphere. Besides removing volatile compounds that alter the taste and the odor of the oil, pigments and other contaminants may be removed as well.

All these traditional refining processes involve the use of chemicals that affects their environmental impact or require high temperatures that are not suitable for thermolabile compounds.

Environmental-friendly alternative options have been proposed, e.g., the use of activated carbon to adsorb some contaminants or enzymatic degumming to remove phospholipids without altering the quality of the oil [50,65,66]. Supercritical fluid technology has been used to remove some undesired compounds like free FAs or toxic compounds (e.g., dioxins), and to degum or bleach the oil as well [50,51].

It is noteworthy that the refining process may lower the content in antioxidants that should be always monitored to evaluate if further addition is necessary [60,67].

## 8. Fish Oil Transesterification

Different purification techniques may require to be preceded by different processing procedures, but a typical way to improve purification yield is oil transesterification [23].

FAs are naturally present in fish oil in the form of triacylglycerols with ω-3 PUFAs preferentially bound at the sn-2 position of the glycerol backbone because this position offers higher stability against oxidation [51,52,68]. Fractionation of intact TAGs would lead to oil containing also the other fatty acid linked to the glycerol backbone (concentrations of up to ca. 30% EPA and DHA) [20]. To achieve a high grade of purity it is necessary to free the FAs from the glycerol moiety and this is usually done by transesterification of crude oil with an alcohol to give fatty acid monoesters. The preparation of such esters is commonly carried out by lipid analysts to convert fatty acid components into esters to be analyzed by GC. Fatty acid methyl esters (FAMEs) are usually preferred for analytic purposes while for food application ethanol is preferred because it is generally recognized as safe (GRAS) by the U.S. Food and Drug Administration (FDA).

**Figure 4:**
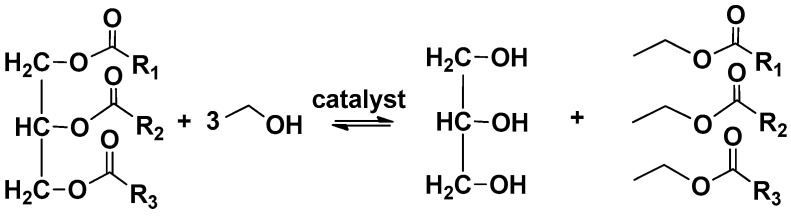
Transesterification reaction of a triglyceride with ethanol to give three fatty acid ethyl esters (FAEEs).

TAG transesterification consists of a succession of three reversible reactions, each one releasing a FA ester molecule. TAGs are firstly converted to diglycerides (DAGs) that are in turn converted to monoglycerides (MAGs) and finally to glycerol [69,70]. Overall, three fatty acid ethyl esters (FAEEs) and one glycerol unit are produced from one TAG (Figure 4). About 10 w/w% of the total product is constituted of glycerol that can be purified and converted into value-added chemicals [15].

While a wide range of strategies is possible to carry out transesterification for analytical purposes [71], on a large scale strong acid or alkaline catalysts are usually preferred. However, the choice should be made aiming at reducing costs, energy utilization, and/or waste generation while keeping reaction yield high, and innovative techniques such as enzymatic catalysis are gaining interest.

Acid catalysis: an acidic catalyst allows both esterification and transesterification under similar conditions. In the first case, a carboxylic acid is protonated to give an oxonium ion that reacts with the alcohol to give in turn an intermediate that can lose a proton and form an ester. In the case of transesterification, a protonated ester reacts with the alcohol forming an intermediate that dissociates into the desired product. Both esterification and transesterification need an excess of alcohol to shift the equilibrium toward esters formation because each step is reversible. Besides, water should be excluded because it is a stronger electron donor than alcohol and hindered the progress of the reaction. Some commonly used reagents are alcoholic solutions of hydrogen chloride or sulfuric acid [71].

Alkaline catalysis: an alkaline catalyst allows transesterification but not esterification. In an alkaline medium, esters form an anionic intermediate that can form the new ester. Carboxylate anions do not undergo a nucleophilic attack by alcohols because of their negative charge. Again, an excess of alcohol is needed to displace the equilibrium and the presence of water should be limited because it causes the intermediate to dissociate into the free acid. Sodium or potassium ethoxide complete the reaction extremely quickly but potassium hydroxide is often used because it is easier to handle. When the reaction is completed, a diluted acid can be added to neutralize the base [71].

A two-steps acid–base catalyzed transesterification is also possible, allowing the processing of oils with high content in free acid and moisture: during the first step free fatty acids are esterified while during the second step transesterification takes place [72].

On an industrial scale, alkaline catalysis is one of the most frequently used methods because of its low cost. Several parameters should be considered when optimizing the reaction condition including the ethanol/oil molar ratio, the catalyst concentration, the water content in ethanol, the reaction temperature time.

When this reaction is carried out on fish oil, three aspects are essential [42,73,74]:

- Water content must be lower than 0.3% to limit the formation of free fatty acids. Freeze-drying or anhydrification by anhydrous sodium sulfate may be used to lower water content.
- Free fatty acids content should be lower than 1 mg KOH/g because they do not react under these conditions and tend to form soaps.
- Temperature and time should be tuned to avoid double bond isomerization and migration.

The use of ethanol makes the reaction more challenging if compared with the transesterification carried out via methanolysis because the production of a more stable emulsion makes the FAEEs purification difficult, but the use of absolute ethanol strongly improves the separation [3].

After the reaction, FAEEs are purified through different extraction steps using water or aqueous acid solution. An initial neutral washing eases the separation reducing the content in soaps, limiting the formation of an emulsion. Diluted hydrochloric acid or citric acid are commonly used, but being pollutive and corrosive may be effectively substituted by water saturated with CO_2_ [75].

Chemical transesterification is well established but poses serious environmental problems. Enzymatic methods have been proposed to save energy and minimize thermal degradation of ω-3 FAs and several lipases are commercially available for this purpose. Lipases are widely used because of their versatility, being able to catalyze both hydrolysis and transesterification reactions under mild conditions, limiting the use of organic solvents. Moreover, these enzymes can be immobilized in appropriate supports and reused providing an economic benefit. The production of FAEEs can be performed in one step or two-steps procedures. In the one-step reaction, the oil is directly transesterified to FAEEs while in the two-steps hydrolysis is followed by esterification.

Ethanolysis products can be concentrated and, depending on the supplement producer’s requirements, either reconverted into TAGs or used to produce 2-MAGs enriched in ω-3 FAs [63].

Since several lipases are active and stable in scCO_2_, it is possible to use it for favoring the miscibility of the reactants, and limiting the use of organic solvents. The enantioselectivity of the enzyme can be improved by tuning pressure and temperature [63].

## 9. Fractionation Techniques

By properly selecting the raw material, it is possible to obtain fish oil with a content of DHA and EPA in the range of 10–25% by weight but since commercial demand calls for content from 60 to 90%, a separation step is needed. FAO standards distinguish between “concentrated fish oil”, containing from 35 to 50 w/w% of fish oil, and “highly concentrated fish oil” (more than 50 w/w% of DHA and EPA as the sum of the two FAs) [42,44].

Omega-3 fatty acids concentration can be performed using different technologies, including molecular distillation (MD), urea precipitation, enzymatic enrichment, and supercritical fluid techniques. A combination of two or more of them is often needed because the raw material is extremely heterogeneous and contains different undesirable compounds. The most suitable method depends on raw material composition, target purity, selectivity, stability, and environmental considerations. Fractionation of FAEEs cannot always be carried out by conventional processes, because of thermal degradation of polyunsaturated chains, or the presence of residual solvent in the final product [60,76]. Other principles should be taken into account by a sustainable fractionation process, including avoidance or reduced use of toxic or polluting reagents and minimization of energy requirements.

Urea fractionation can separate FAs according to their degree of unsaturation [77]. Triglycerides are hydrolyzed to give free fatty acids that are treated with urea and ethanol. Urea can form solid-phase complex molecules with linear alkyl chains like SFAs and MUFAs. These complexes precipitate under cooling (ca. 20 °C) and are removed by filtration, leaving a liquid fraction enriched in PUFAs [1,20,78,79]. This technique is generally considered inexpensive due to the low cost of reagents, and ecofriendly because the whole process only requires little compounds that are generally recognized as safe (GRAS) by FDA. This method allows a 60% recovery and can be used as a preliminary purification step before other purification processes. Some concerns have been raised by U.S. FDA in 1999 due to the formation of ethyl carbamates (EC), a potent animal and human carcinogen, from the reaction of urea and ethanol [80]. Proper washing procedures, however, seem to remove EC from the product.

Winterization separates fatty acids based on their different melting points: controlled cooling induces the crystallization of saturated and monounsaturated fatty acids, removed by filtration. This process can be applied before other stages to reduce the complexity of the lipid mixture.

Currently, the most utilized method to concentrate ω-3 FAs at an industrial scale is probably molecular distillation (MD), also known as short path distillation. Compounds are separated according to their vapor pressures under high vacuum conditions (10^–3^ bar), forcing the molecules to travel a pathway that is shorter than their mean free path. FAEEs have a lower boiling point than triglycerides (ca. 450 °C vs. >900 °C) and this allows an easier separation [64,81]. MD is widely used on an industrial scale, nonetheless, yield and selectivity are quite low (between 55 and 70%) if compared to the market demand for highly concentrated oils (≥85%) [3,63,64]. Moreover, despite the short time of operation, this technique operates in the range of 140–170 °C, constituting a risk for thermolabile compounds.

To avoid problems related to PUFAs thermolability enzyme-catalyzed methods allowing mild operating conditions and chemicals have been developed [61]. Some lipases can discriminate against ω-3 fatty acids and this ability has been ascribed to the steric hindrance provided by the double bonds in the active site of the enzyme. Moreover, these methods can carry out the ω-3 FAs enrichment dealing directly with intact TAGs, skipping the transesterification step. Microbial lipases hydrolyze TAGs into fatty acids, acylglycerols, and glycerol. Free fatty acids are then esterified with simple alcohol or glycerol [67].

Supercritical fluid fractionation (SFF) using scCO_2_ is an interesting potential substitute for other more toxic or environmentally hazardous organic solvents that are also difficult to dispose of. Since its supercritical conditions are easily reached, it is already an established system for the extraction and purification of pharmaceutical and food products [82].

Short-chain FAEEs are more soluble in scCO_2_ than long-chain FAEEs and this difference in solubility serves as a basis for the fractionation. In a typical separation process, the liquid FAEE mixture flows downwards, while the scCO_2_ flows upwards (in counter-current) solubilizing short-chain FAEEs that exit the column from the top, giving an enrichment of EPA and DHA in the raffinate (the liquid remaining inside at the bottom of the column). The lighter phase is partly condensed and recycled after recompression (reflux). When the system is brought back to atmospheric pressure, the oil is completely separated from the carbon dioxide, leading to the complete removal of the solvent [76,83].

Phase equilibria data and thermodynamic and kinetic models are essential to a proper separation process because phase equilibrium data and suitable equations of state allow one to predict miscibility regions under different conditions and thus reduce the experimental effort. Actually, due to the high cost of investment for the construction of pilot plants, research often focuses on constructing models, performing simulations of equilibrium stage separations, and comparing their outputs with experimental data.

An interesting example of process design, modeling, and economical feasibility of fish oil fractionation by supercritical fluids was presented by Fiori et al., pointing out how the whole fractionation can be performed at an industrial scale utilizing relatively mild conditions (temperature below 100 °C) [83]. Three indicators were considered: yield, recovery, and quality (85 wt % of C20 and C22). The authors modeled an SFF process using commercial software (Aspen Plus^TM^) and used this model to evaluate the effects of several parameters, including the feed ratio, reflux ratio, temperature, and pressure. Moreover, operation costs were optimized, considering the investment cost and economic feasibility for different plant sizes and pointing out the advantages of working with large capacity plants.

Supercritical fluid chromatography proved to be a valuable and effective method to reach high enrichment levels. Tuning temperature and pressure (typically from 90 to 300 bar and from 298 to 337 K) it is possible to modulate scCO_2_ solvent properties, possibly using ethanol to improve the solubility of more polar compounds. Both packed or capillary columns can be used, and appropriately choosing stationary phases and operation conditions, highly pure ω-3 FAs concentrates can be obtained also at a large scale suitable for food and pharmaceutical applications [23]. Reversed phases are preferred to normal phases because the latter can only poorly retain non-polar fatty acid esters. Octadecylsilane-grafted silica and alkali-treated alumina stationary phases give a better resolution. A coupled system SFE-SFC can be used to remove polar FFAs or oxidation products [63].

In a study by Alkio et al. [23], the technical and economic feasibility of an ester concentrate from tuna oil is presented. In industrial chromatography, the increased amount in injection volume causes a decrease in column efficiency and high loads in lower yields. Some drawbacks are the high cost of investments that in this case are also used to special coating materials used in stationary phase manufacturing, nonetheless, a large-scale SFC plant is already operating producing fatty esters concentrate at >95% purity.

Even if MD is the most used method by now, SFF and SFC could replace it because of their higher selectivity and lower operating temperatures, which minimizes the risk for oxidation. These observations are also corroborated by favorable economic analyses [63]. The main obstacle to a large-scale application is that SFF processes should be designed for each specific application, lacking a ready availability.

## 10. Omega-3 Fatty Acids Formulations and the Encapsulation Process

Omega-3 fatty acids are highly lipophilic molecules whose absorption and bioavailability are limited and require a proper delivery system to be improved. Furthermore, their susceptibility to oxidation causes the formation of degradation products that may be toxic and decrease the acceptance of consumers not only because of safety concerns but also because of the unpleasant smell and taste of some of these products their selves. This factor is of non-secondary importance since fishy burp is one of the main reasons consumers limit their ω-3 FAs intake [46]. A proper formulation allows overcoming these problems as well [84],

Omega-3 FAs can be present in several forms in supplements, including:

- Natural TAGs;
- Ethyl esters;
- Re-esterified TAGs enriched in ω-3 Fas;
- Free fatty acids;
- Phospholipids.

The extent of the absorption of the different chemical forms of EPA and DHA is a debated issue. Omega-FAs absorption seems to decrease in the following order: fatty acids, triglycerides, and ethyl esters. However, different results in different studies may depend on the nutritional matrix and the study duration [85]. Human ethyl esters processing involves the conversion to triglycerides delaying their release to the bloodstream. The conversion of ω-3 ethyl esters back into triglycerides form raises from the observation that ω-3 FAs absorption seems to be faster and higher when TAGs are consumed [63,85].

This reaction can be carried out by enzyme-assisted methods (e.g., using lipases): ethyl esters are converted to free fatty acids that are in turn esterified with glycerol to produce highly concentrated triglycerides. The use of specific lipases reduces the formation of byproducts, allows mild reaction conditions, and limits the need for organic solvents. Ethanol is produced during the reaction and should be removed because it is toxic to the enzyme and could inhibit the reaction shifting the equilibrium towards the reagents. Additionally, in this case scCO_2_ can be used as a solvent at low temperatures and in an oxygen-free atmosphere.

Phospholipids are usually found in fish roes, krill oil, and to some extent in oily fish and have recently received increasing attention for their health benefits. While TAGs and ethyl esters are hydrophobic, phospholipids are hydrophilic and this affects their physical–chemical properties. PLs and TAGs are equivalently absorbed in the small intestine but their delivery in different organs may be different.

The selective delivery and the controlled release of an active compound increase its efficacy and are favored by the control of particle size and encapsulation in a suitable polymer. Several formats are possible: chews, gummies, liquids, gels, and capsules [63,64]. Encapsulation is an essential step in supplement production and it is commonly used because not only does it allow the release of the ω-3 FAs directly in the intestine but also protects lipids from oxidation, extending shelf-life and masking taste and smell [84].

Micronization techniques are often used to downsize particles for pharmaceutical and food application to micron (and more recently, nano) level, improving material properties like solubility, flavor release, or mouthfeel [86]. Smaller particles are associated with higher rates of dissolution, increased bioavailability, and lower dosages.

Two approaches are possible: larger particles may be reduced to a smaller size (“top-down”) or microparticles can be built from molecules in solution (“bottom-up”). Classical top-down methods include homogenization and grinding, while techniques involving supercritical fluids usually belong to the second kind of approach.

Encapsulation of ω-3 FAs is currently achieved by different techniques. Among classical methods, spray-drying and coacervation are widely used also on a large scale. The selection of the more appropriate method depends on the interaction of the solvent with the compound of interest, the desired morphology, the particle size distribution, and solvents used (if any) [87]. The choice of the coating material is critical and closely connected to the method of encapsulation: it should have low viscosity, good film-forming proprieties, and neutral organoleptic characteristics. Moreover, it should ensure the preservation of the bioactive core from degradation [88]. When dealing with spry drying and coacervation, protein or polysaccharides are typically used as shell materials [89].

Other parameters to be evaluated are the encapsulation efficiency and the residual solvent concentration. Encapsulation efficiency is the proportion of fish oil encapsulated with respect to the theoretical content and is essential to the economic feasibility of the process. It also affects the acceptability of the product, because external oil is more susceptible to oxidation, impairing its organoleptic features. It can be determined by GC quantifying the fatty acid previously methylated. Residual solvent concentration should comply with food and drug regulations.

Traditional micronization and encapsulation techniques do not usually allow a high level of control of particle size distribution. Moreover, they often use toxic and polluting solvents and need high temperature, unsuitable for thermolabile compounds. For example, spry drying requires high temperatures that may cause oil deterioration [84]. Another important factor is that residual oil adherent to the capsule surface can easily oxidize altering the organoleptic properties of the supplement.

Other techniques have been proposed to overcome these problems. Freeze-drying of emulsions minimizes oxidative degradation of ω-3 FAs by removing oxygen and operating at low temperatures but it is more costly than spray-drying and it is not suitable for industrial applications [89]. Encapsulation of omega-3 FAs in the lipid vesicular system on the micro- and nano-scale provides good stability but the high costs and the use of organic solvents (e.g., chloroform) made this method not sustainable [89,90]. More information about the microencapsulation techniques of ω-3 fatty acids can be found in a review written by Kaushik and colleagues [89].

Supercritical fluids reduce the use of organic solvents, preserve the lipids from oxidation, and allow producing not only micro- but also nanocapsules at low temperature and under an inert atmosphere. Three main SFC micronization variants do exist:

- Supercritical solvent precipitation: the fluid is used to solubilize non-polar active ingredients (e.g., essential oils and non-polar small compounds). An example is the expansion of supercritical solutions (RESSs), with both the active ingredient and the coating at high pressure are dissolved in sCO_2_. The pressurized solution is rapidly expanded through an orifice. Due to supersaturation, the coating material desolvates and deposits around the active compound forming the capsules [87]. This method is limited by the scarce solubility of most material in scCO_2_ but it is interesting because it is completely solvent-free and allows particles with a size <1 μm [91].
- Supercritical antisolvent precipitation: the active ingredient is dissolved in an organic solvent and the supercritical fluid is used as an antisolvent to induce its precipitation, by extracting the organic solvent from the solution. Examples are supercritical antisolvent (SAS) and supercritical fluid extraction of emulsions (SFEE). SAS is used to produce highly bioavailable capsules of hydrophobic compounds or hydrophilic compounds that can be homogeneously dispersed in an organic solvent. The solute is dissolved (or dispersed) in a suitable solvent then the addition of the supercritical fluid causes a reduction in solubility and the consequent precipitation of micro- and nanoparticles [91]. In the SFEE technique, to encapsulate lipophilic compounds a stable oil-in-water emulsion is formed. The organic solvent is rapidly extracted by the counter-current flow of scCO_2_ that induces the active ingredient and the polymer precipitation [87]. This technique was used to encapsulate fish oil rich in ω-3 PUFAs using polycaprolactone (PCL) as a coating polymer and reaching 8 nm particle size [84]. Among its advantages, it is easily scalable for industrial applications using countercurrent packed columns and it allows the production micro- and nanoparticles with controlled size and morphology. Moreover, the control of the particle morphology is possible on a wide range of sizes, from nano to micro [88]. The encapsulation of fish oil by a supercritical antisolvent process using hydroxypropyl methylcellulose (HPMC) as a polymer is reported as suitable for the industrial application [88].
- Supercritical fluid as a solute: this technique exploits the high diffusivity of scCO_2_ and its low viscosity to make it diffuse into solutions, polymer melts, and fatty acids. An example is particles from gas saturated solutions (PGSSs): a compressed fluid is dissolved into a solution that is then expanded through a nozzle into a spray chamber at atmospheric pressure. Due to the expansion, the CO_2_ is removed by evaporation, and the temperature decreases, causing the polymer solidification. This technique is suitable for lipids because they melt at a mild temperature [86,87].
- Methods based on drying technology: in these techniques, the supercritical fluid is used to dry aqueous solution and wet samples avoiding high temperatures that may damage thermolabile compounds. An aqueous solution is sprayed into an excess of the supercritical fluid. The water is broken up into tiny droplets that are easily removed from the fluid flow.

Complete reviews of the currently available options and applications of sCO_2_ for micronization and encapsulation were published by Soh et al. and by Dhiman et al. [86,87], which provided more detailed information on the techniques and the mechanism behind them.

Antioxidants can be added to protect lipids against oxidation. Some possible additives are butylated hydroxyanisole (BHA), butylated hydroxytoluene (BHT), or a combination of fatty esters derivatives of ascorbic acid and metal chelators. The increased preference for natural antioxidants prompted the research for alternatives that include polyphenols or tocopherols [63].

## 11. Fish Waste and Sustainability

Global fish production is estimated to have reached around 179 million tons in 2018 distributed as follows: ca. 156 tons for human consumption and ca. 22 million tons for animal feed and fish oil production [92].

Fish processing and farming can generate considerable amounts of leftovers and byproducts that negatively affect the environment. Cleaning the equipment and flushing the offal away spraying water generate additional effluents. Fish waste is often used as a low market product (e.g., animal feed or fertilizers), directed to a landfill, or discharged back into the sea altering the habitat with an increase in nutrients and oxygen consumption.

Some of this waste can be efficiently converted into marketable high-value products, representing a more interesting option than their non-utilization or disposal towards open waters or landfills.

According to a recent report by FAO, marine fishery resources are declining but the number of wild species utilized for fish meal production decreased from over 30 million tons in 1994 to about 18 million tons in 2018 due to the adoption of good waste management practices and the aquaculture industry [92].

Around 918,000 tons of fish oil is extracted every year and the amount deriving from waste should increase from 40 to 45% [92]. Due to the expanding demand for ω-3 FAs concentrates, oil extraction from fishery waste represents an appealing alternative option for their production, especially if considering that fish oil does not compete with food resources [93]. On these bases, the development of an industrial-scale concentrates production route that allows reducing both costs and environmental impact preserving DHA and EPA properties is essential.

Most of the works discussing how to implement a sustainable model in the oleochemical industry focus on biodiesel production but these options are not always applicable to marine oil due to thermal degradation [94,95].

Fish oil for food consumption usually needs to be refined and this treatment requires a massive amount of chemicals, including phosphoric acid and caustic soda 94]. The use of these chemicals and the high number of processing stages, causes oil losses, an increased risk in oil oxidation, and detrimental environmental impact [64].

Energy consumption is another essential aspect that should be considered when evaluating the sustainability of a process. In fish processing, this is mainly used for heating, cooling, and operating electric apparatuses. Depending on how this energy is produced, its consumption may contribute to fossil fuel depletion. Saturated FAEEs may be used as biodiesel to improve the process profit and close the circular economy model. The use of renewable energy sources would constitute an interesting approach to improve the sustainability of the recovery process [14].

In 2017, Fiori et al. published a case study of a biorefinery strategy for fish waste valorization into ω-3 FAs concentrates, protein for fish farming applications, and short-chain FAEEs for fuel production [3]. In general, technologies based on supercritical scCO_2_ can be used in different phases of the waste processing (extraction, refining, transesterification, concentration, and encapsulation), provide conditions that reduce the risk of oxidation (low temperature and inert atmosphere), show good solvent and mass transport properties, and offer the possibility of recycling the fluid by decompression and recompression cycles. Another advantage is that CO_2_ is gaseous at room temperature and pressure, easily providing solvent-free products. The favorable and tunable properties of supercritical CO_2_ make it a very and sustainable alternative to other processing methods.

Another interesting example has been recently published reporting the implementation of sustainable options in the production of ω-3 FAs concentrates from *Thunnus albacares* waste and its benefit from an environmental, economic, and social point of view [94]. The manuscript by Monsivais et al. shows how alternative options at different levels can have a significant effect on the final environmental or social impact of the product, thus generating several ways to improve the circular economy model. In this context, the most profitable design is not always the optimal-sustainable and, therefore, a maximum economic advantage does not always guarantee a circular economy achievement.

Different interactive factors are contributing to an effective reduction of waste reduction and different solutions should be adopted in different contexts. Besides, a detailed evaluation of the advantages and disadvantages should always be carried out because the best option is not always evident. For example, while aquaculture has provided a source of food and income, contributing to reducing and reusing waste material, on the other hand, fish farming requires the majority of fish body oil and affects coastal environments and biodiversity because of the release of organic waste and chemicals used for medication [6]. Future industry development must not set aside social issues and provide quality and accessible products to the communities that represent the workforce and mankind’s progress cooperatively.

Governments should proactively act in building effective policies and regulations, supporting innovative solutions with adequate infrastructure and services. Their action would fall within the global commitment demonstrated by “The 2030 Agenda for Sustainable Development”, a set of 17 interconnected goals, adopted by all United Nations Member States in 2015.

The fisheries and aquaculture sectors are related to SDG 14, “Conserve and sustainably use the oceans, seas and marine resources for sustainable development”. However, the proper management of fish waste and its contribution to providing food, nutrition, and employment, is also strictly related to the SDG 12, entitled “Responsible consumption and production”.

Passing on to the economic side of the recovery, the price of fish oil reached 2800 USD/ton in 2015 while in the same year the ω-3 FAs market worth 28 billion euros (ca. 30 billion USD) [13,64]. Provided that the valorization process is carried out following sustainable practices, the exploitation of fish waste is a promising tool to match the increased market demand maximizing the economic potential of this valuable resource. In compliance with circular economy and zero waste principles, fish waste represents an invaluable source of products: ω-3 FAs concentrates, MAG and DAG that can be used as emulsifiers, glycerol for the production of other valuable chemicals, FAEEs other from DHA and EPA as liquid biofuel, fish bones as a source of minerals, collagen, and gelatin. An informative synoptical presentation of various fish waste uses is given by Arvanitoyannis and Kassaveti in a review of 2008 [6].

## 12. Conclusions

Fish oil-derived dietary supplements are widely marketed across the world and this urges the development of responsible strategies to utilize resources that are renewable but not infinite. The efficient minimization and exploitation of fish waste and its transformation into high-value products is an attractive solution both from an environmental and economic point of view.

Despite undeniable signs of progress, great room for improvement exists, making essential the promotion of sustainable practices sustained by technological innovation and government policies. Further research is necessary to obtain highly pure, stable, safe, and bioavailable ω-3 concentrates, while clear communication of improvements in sustainability along with the safety of products obtained from waste is essential to consumers’ acceptability. Waste should not be considered less valuable than the fish itself but a precious and profitable resource capable of bringing health, social, economic, and environmental benefits.

## Figures and Tables

**Table 1:** Oxidation quality parameters ^1^.

Acid Value	≤3 mg KOH/g
Peroxide Value	≤5 meq of active oxygen/kg oil
Anisidine Value	≤20
ToTox	≤26

^1^ Method of analysis and sampling can be found in “Recommended Methods of Analysis and Sampling (CXS 234-1999)” [45].
